# Nonlinearity Correction in OFDR System Using a Zero-Crossing Detection-Based Clock and Self-Reference

**DOI:** 10.3390/s19173660

**Published:** 2019-08-22

**Authors:** Shiyuan Zhao, Jiwen Cui, Jiubin Tan

**Affiliations:** 1 Center of Ultra-Precision Optoelectronic Instrument, Harbin Institute of Technology, Harbin 150080, China; 2 Key Lab of Ultra-Precision Intelligent Instrumentation, Harbin Institute of Technology, Ministry of Industry and Information Technology, Harbin 150080, China

**Keywords:** optical fibers, Rayleigh scattering, optical frequency-domain reflectometry, strain measurement

## Abstract

Tuning nonlinearity of the laser is the main source of deterioration of the spatial resolution in optical frequency-domain reflectometry (OFDR) system. In this paper, we develop methods for tuning nonlinearity correction in an OFDR system from the aspect of data acquisition and post-processing. An external clock based on a zero-crossing detection is researched and implemented using a customized circuit. Equal-spacing frequency sampling is, therefore, achieved in real-time. The zero-crossing detection for the beating frequency of 20 MHz is achieved. The maximum sensing distance can reach the same length of the auxiliary interferometer. Moreover, a nonlinearity correction method based on the self-reference method is proposed. The auxiliary interferometer is no longer necessary in this scheme. The tuning information of the laser is extracted by a strong reflectivity point at the end of the measured fiber. The tuning information is then used to resample the raw signal, and the nonlinearity correction can be achieved. The spatial resolution test and the distributed strain measurement test were both performed based on this nonlinearity correction method. The results validated the feasibility of the proposed method. This method reduces the hardware and data burden for the system and has potential value for system integration and miniaturization.

## 1. Introduction

An optical frequency-domain reflectometry (OFDR)-based distributed sensing system was initially proposed by Froggatt et al. in 1998 and utilized in distributed disturbance measurements such as strain or temperature measurements because of its high spatial resolution and sensitivity [1,2,3]. In an OFDR system, the interference signals are collected as a function of the optical frequency of a tunable laser source (TLS). A fast Fourier transformation (FFT) is then used to convert this optical frequency-domain information to a desired spatial information, where it is required that the interference signals are sampled at an equal interval of optical frequencies [4]. However, any frequency tuning nonlinearity of a TLS gives rise to a non-uniform sampling interval of the optical frequency when the signal is sampled by an equal time interval, which in turn results in spreading of the reflection peak energy, deteriorating the spatial resolution [4].

Two kinds of methods were developed in recent years to solve the problem of the laser tuning nonlinearity. The first involves utilizing an auxiliary interferometer to produce an external sampling clock as a data acquisition trigger [5]. Although this method occurs real time and does not need post-processing, the maximum measurement length is limited by the time delay of the auxiliary interferometer in order to satisfy the Nyquist law [6]. This limits the measurement range of the system. Some researches proposed in-phase quadrature detection (IQ), such as the 3 × 3 coupler [7] or optical hybrid receiver [8], which can double the detection length. However, in these schemes, a complex operation in the demodulation is introduced, which would decrease the real-time demodulation of the system.

The second method to correct the tuning nonlinearity is to perform post-signal processing after acquiring OFDR data. Normally, this technique involves acquiring an auxiliary interferometer signal along with the OFDR signal and extracting the TLS phase information from the auxiliary interferometer signal, compensating nonlinearity in the OFDR signal using a correction algorithm [4]. One of the algorithms involves the resampling technique that resamples the main interference signals with an accurate equidistant optical frequency grid based on the optical frequency information of the TLS using interpolation algorithms. Badar et al. proposed a self-correction scheme in which only one detector is contained in the measurement system. In their proposed scheme, an intentional beating signal is introduced at the beginning of the OFDR spectrum, which is treated as an auxiliary interferometer to acquire tunable laser phase information for post-signal processing [9]. However, two drawbacks exist in their scheme. One lies in the fact that an extra delay for the intentional beating signal is introduced in the system, which increases the instability. Another lies in the fact that the intentional beating signal is at the beginning of the spatial domain. This design makes the optical path difference (OPD) of the main interferometer much longer than that of the auxiliary interferometer. Therefore, much interpolation is implemented, which increases the probability of false information, and also significantly increases the data volume. Moreover, the intentional beating signal occupies one segment at the low-frequency position, which sacrifices a part of the effective measurement range.

In this paper, tuning nonlinearity correction methods in an OFDR system were developed from the aspect of data acquisition and post-processing. On one hand, a new hardware-based method for real-time sampling is presented, which was implemented by designing an external clock to provide triggers at zero-crossing positions with uniform frequency spacing. The limited measurement range, which is equal to one-half of the OPD of the auxiliary interferometer in the conventional OFDR acquisition mode, can double and extend to the same length with the OPD of the auxiliary interferometer. On the other hand, the nonlinearity correction based on a single interferometer and self-reference is demonstrated. The tuning information of the laser is obtained from a PC connector at the end of the measured fiber. In this case, no extra delay fiber is needed because the fiber to be measured also plays the role of the optical path of the PC-constituted interferometer. Additionally, since the PC connector is at the end of the measured fiber, the OPD of the PC-constituted interferometer is longer than that of the main interferometer. That means that the beating frequency of the PC connector is larger than that of the reflectivity points (served as the sensing part) before the PC connector. Therefore, in the algorithm demonstrated later, substantial phase subdivision is not needed to resample the interferometer fringe signal. This makes the effect of the nonlinearity correction more stable.

The rest of this paper is organized as follows: Section 2 and Section 3 describe two approaches for correcting nonlinearity of the laser. The first is the external clock based on zero-crossing detection. The circuit design and scheme are demonstrated. This method is validated by the spatial resolution test and maximum measurement distance. The second is nonlinearity correction using self-reference. The principle of the method is described. The OFDR trace is analyzed, and the distributed strain is measured based on the self-reference method. Section 4 concludes this paper and gives a brief comparison of the two methods proposed in this paper.

## 2. Method 1: External Clock Based on a Zero-Crossing Detection

### 2.1. Method Description

Ignoring the phase noise of the laser, the interference pattern of the auxiliary interferometer with a Mach–Zehnder scheme can be written as

(1) $$I_{a}=\cos[2\pi \gamma (t)\tau_{a}t+\phi_{0}].$$

In Equation (1), γ(t) is the tuning speed of the laser, $\tau_{a}$ is the time delay of the Mach–Zehnder interferometer, and $\phi_{0}$ is the initial phase. Equation (1) is equal to 0 when the phase is

(2) $$2\pi \gamma (t)\tau_{a}t+\phi_{0}=\frac{\pi }{2}+k\pi .$$

In Equation (2), *k* is an integer.

According to $t=\frac{v(t)}{\gamma (t)}$, where v(t) is the optical frequency, Equation (2) can be written as

(3) $$2\pi v(t)\tau_{a}+\phi_{0}=\frac{\pi }{2}+k\pi \;(k=1,2,\ldots ).$$

Then, the optical frequency at the zero-crossing points can be expressed as

(4) $$v(t_{k})=\frac{k}{2\tau_{a}}+v_{c}.$$

In Equation (4), $v_{c}$ refers to a constant. It can be seen from Equation (4) that the optical frequency increment is equal and related to the OPD of the auxiliary interferometer at each zero-crossing point.

Next, we analyze the signal of the main interferometer. The interference intensity, which is interfered by the reflectivity point *D* on the measured fiber and the local light from the reference arm in the main interferometer, can be written as

(5) $$I_{D}=\cos[2\pi v(t)\tau_{D}+\phi_{d}].$$

In Equation (5), $\tau_{D}$ is the time delay between point *D* and the local light from the reference arm, and $\phi_{d}$ is the initial phase. The zero-crossing positions of the interferometer pattern of the auxiliary interferometer can be used to resample the beat signal expressed as Equation (5). When resampling the interference signal $I_{D}$ with an interval of $\Delta v=\frac{1}{2\tau_{a}}$ as indicated in Equation (4), the resampled signal can be written as

(6) $$I_{D,M}=\cos[\pi \frac{M}{\tau_{a}}\tau_{D}+\phi_{d}]\;(M=1,2,\ldots ).$$

The trigger frequency of the auxiliary interferometer is $f_{a}=2\gamma \tau_{a}$. The beating frequency of the main interferometer is $f_{D}=\gamma \tau_{D}$. To satisfy the Nyquist criterion, it is necessary that $f_{a}>2f_{D}$ and $\tau_{a}>\tau_{D}$. Therefore, the measurable distance could reach the distance of the auxiliary interferometer. However, most commercial data acquisition cars (DAQs) can only sample at the rising or falling edge if using the external clock mode. This results in the measurable distance being limited to half the OPD of the auxiliary interferometer.

### 2.2. Hardware Implementation

As demonstrated by the principle above, the core task lies in designing a circuit which can detect all the zero-crossing points of the interferometer signal of the auxiliary interferometer (AI). Considering the tuning nonlinearity of the laser, the sinusoidal signal has a frequency fluctuation centered at its nominal beating frequency. The requirement for the circuit is high speed and low time delay. Furthermore, since the nominal beating frequency depends on the tuning speed of the laser, the maximum frequency of the AI signal is determined by the required measurement speed of the system. In our system, the maximum frequency of the AI signal is designed to be 20 MHz. That means that the maximum input frequency of the circuit for zero-crossing detection is 20 MHz. The total time delay of the circuit should be less than half a period of the AI signal. Under this rule, the trigger signal of the circuit can be considered to track and reflect the changing-frequency AI signal with success. The half-period is 25 ns when the frequency is 20 MHz. Therefore, it should result in the final trigger signal having a total time delay less than 25 ns on the consideration of the device selection for the circuit.

**Figure 1:**
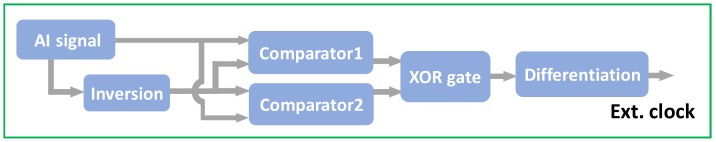
Circuit scheme of the zero-crossing detection for the auxiliary interferometer (AI) signal.

**Figure 2:**
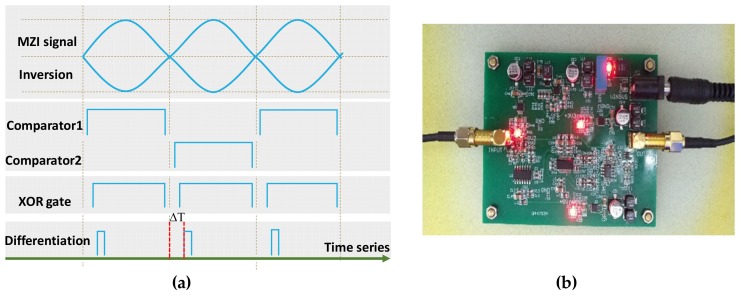
(**a**) Time-series analysis for each node on the circuit; (**b**) photograph of the circuit board based on the proposed zero-crossing detection scheme.

Figure 1 shows the circuit scheme of zero-crossing detection for the AI signal. Figure 2a gives the signals at each node. The AI signal is inversed firstly. Then, the original AI signal and its inversion signal are sent into the two comparators. The output of the two comparators goes through a high level every half-period of the AI signal. Then, the output of the comparators is sent to an XOR gate, after which the square wave appears at each half-period of the AI signal. The differentiation unit is used to convert the square wave to a narrow pulse. The narrow pulse serves as the trigger signal. The rising edge of the trigger signal appears after the zero-crossing of the AI signal. The time delay ΔT depends on the time delay sum of each component delay in the circuit.

### 2.3. Experiment

**Figure 3:**
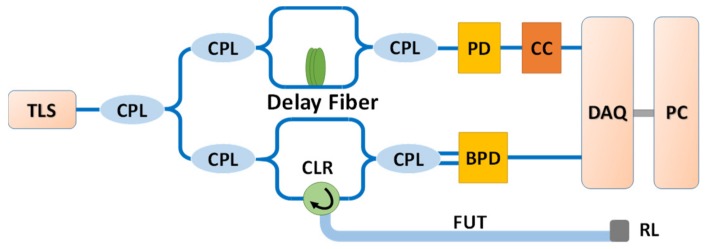
Optical frequency-domain reflectometry (OFDR) system. The auxiliary interferometer is an unbalanced Mach–Zehnder interferometer with a 250-m reference delay fiber. TLS: tunable laser source; CPL: fiber couple; CLR: fiber circular; PD: photo detector; BPD: balanced photo detector; CC: clock circuit; DAQ: data acquisition card; PC: personal computer; FUT: fiber under test; RL: refractive index matching liquid.

A conventional OFDR system is shown in Figure 3. The light from the laser (LUNA, Phoenix 1400 with 3 MHz linewidth) is split into two paths by a 10:90 optical coupler, with 10% light sent to an auxiliary interferometer with a delay fiber of 250 m. The measured fiber has a length of 122 m; thus, its round trip is about 250 m. The end of the fiber is an APC connector immersed in a refractive index matching liquid for the sake of reducing reflectivity. The laser sweeps from 1540 nm to 1560 nm; thus, the two-point spatial resolution Δz of the system is 40 μm calculated by Δz=c/2nΔv, where *n* is the refractive index of the fiber under test (FUT), and Δv is the optical frequency tuning range of the TLS. The customized clock circuit is added after the PD. The sampling mode of the data acquisition card (DAQ) is set to use the rising edge of the external clock as the trigger source.

**Figure 4:**
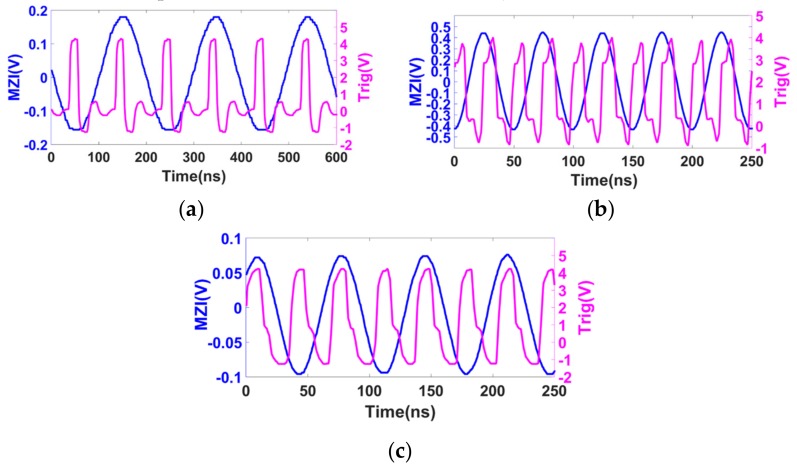
Input–output curve of the clock circuit for zero-crossing detection at different input frequencies. The blue curve is the AI signal and the pink curve is the output trigger signal of the clock circuit. (**a**–**c**) Nominal input frequencies of 5 MHz, 20 MHz, and 12.5 MHz, respectively.

Firstly, the input–output timing sequence of the customized clock circuit was tested. Different tuning speeds were set so that the nominal beating frequencies of the AI signal were different. The beating frequency satisfied $f_{beat}=\gamma \tau$, where γ is the tuning speed with a unit of Hz/s, and τ is the time delay of the auxiliary interferometer. The tuning speeds of 32 nm/s, 80 nm/s, and 128 nm/s corresponded to beating frequencies of 5 MHz, 12.5 MHz, and 20 MHz, respectively. The input–output time sequences are shown in Figure 4. It can be seen that the time delay between the zero-crossing point and its following trigger signal were all within one half-period of the AI signal. Furthermore, this time delay was almost the same and can be considered as constant if the time delay difference of the two comparators used in the circuit is negligent.

**Figure 5:**
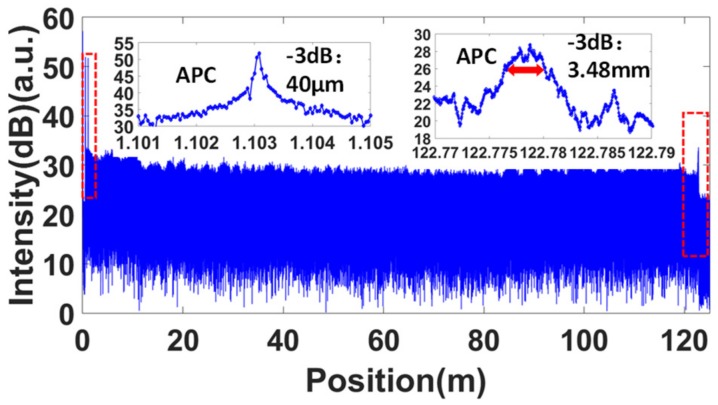
Measured OFDR trace with an APC connector end immersed in a refractive index matching liquid. The first APC connector and the APC at the end of the fiber are shown in the insets. The trace shows a good nonlinearity correction result. The broadened peak of the final APC connector mainly results from the phase noise of the laser and the effect of the refractive index matching liquid.

Then, the OFDR trace was sampled by the DAQ with our customized clock circuit at the condition of 80 nm/s tuning speed. The nominal beating frequency of the AI signal was then 12.5 MHz. The original signal from the main interferometer was fast Fourier transformed to the spatial domain. The result is shown in Figure 5. It can be seen that the measurement length can reach a length equal to the OPD of the auxiliary interferometer. To evaluate the linearity of the system quantitatively, the full width at half maximum (FWHM) of the reflectivity peak of a fiber connector is generally used. The FWHM of the APC connector was 40 μm, which was the Fourier transform-limited spatial resolution [10]. At the end of the fiber, the FWHM of the APC connector decreased to about 3.48 mm. The resolution deterioration mainly came from the increasing phase noise of the laser, which increased with the length, and also from the immersion in the refractive index matching liquid.

## 3. Method 2: Nonlinearity Correction Based on Self-Reference

### 3.1. Method Description

**Figure 6:**
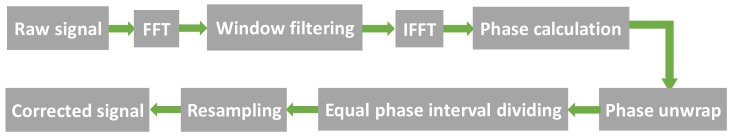
Procedure for the self-reference method for the laser tuning nonlinearity correction.

Figure 6 describes the nonlinearity correction process using the self-reference method. The raw signal in the optical frequency domain was sampled using a fixed sampling rate, then converted to the spatial domain by fast Fourier transformation. A rectangle band-pass filter was applied on the data in the spatial domain. The band should cover the central band of the reference point which may be a PC connector. The reminder of the spectrum was set to zero. After that, the filtered data were inverse fast Fourier-transformed back to the optical frequency domain. After that, the Hilbert transformation (HT) and arc tangent operation were used to extract the phase; then, the phase was unwrapped. The nonlinearity information was obtained. Next, the unwrapped phase was divided into equal-spaced segments with an equal phase internal. These positions were then used to resample the raw signal. Finally, the nonlinearity-corrected interferometer signal was obtained. It should be noted that, in the process mentioned, no interpolation was introduced.

The mathematical operation for the method is demonstrated below.

After filtering, the temporal component of the PC-constituted interference is separated from the original signal. The detected interference pattern interfered by the strong reflectivity point (e.g., a PC connector) with the local light can be written as

(7) $$I_{pc}(t)=\cos[2\pi \gamma (t)\tau t+\xi_{0}]=\cos[2\pi v(t)t+\xi_{0}].$$

In Equation (7), γ(t) is the tuning speed of the laser, τ is the time delay between the PC connector and the local light from the reference arm, and $\xi_{0}$ is the initial phase. The Hilbert transformation (HT) of $I_{pc}(t)$ can be expressed as

(8) $$HT\left\{I_{pc}(t)\right\}=\sin(2\pi \tau v(t)+\xi_{0}).$$

The phase of the interference pattern is, therefore, represented as

(9) $$\phi (t)=2\pi \tau v(t)+\xi_{0}=\tan^{-1}[HT\{I_{pc}(t)\}/I_{pc}(t)].$$

Since the arctangent function maps the phase angle to the range [−π,π], the phase needs to be unwrapped. Derived from Equation (9), the relationship between the change in optical frequency of the laser and the change in phase of the interference pattern can be represented as

(10) $$\Delta v(t)=\frac{1}{2\pi n}\frac{c}{\Delta L}\Delta \phi (t),$$

where ΔL is the OPD between the PC connector and the local light from the reference arm. It can be seen from Equation (10) that the resampled points at each equal optical phase interval represent equal optical internal frequencies. The relationship between the maximum correction range $L_{m}$ and the optical phase interval $\Delta \phi_{\pi }=\pi /K$ (*K* is the subdividing number and is a positive integer) can be represented as

(11) $$L_{m}=K\times L_{pc},$$

where $L_{pc}$ is the distance between the PC connector and the starting position. Generally, a phase increment π or π/2 can meet the nonlinearity correction requirement, and the maximum sensing distance will not be lower than the OPD of the PC connector.

### 3.2. Simulation

To simplify the simulation, we assumed that the optical frequency of the laser satisfied the quadratic function modeled by

(12) $$v(t)=\gamma t+at^{2},$$

where γ is the tuning speed and a is the nonlinearity coefficient [11].

The simulation parameters for the tuning process were as follows: tuning range = 2500 GHz; tuning speed = 5000 GHz/s; sampling rate = 10 MS/s; sampling number = 5 MS; optical frequency deviation at the middle optical frequency = −1 GHz.

**Figure 7:**
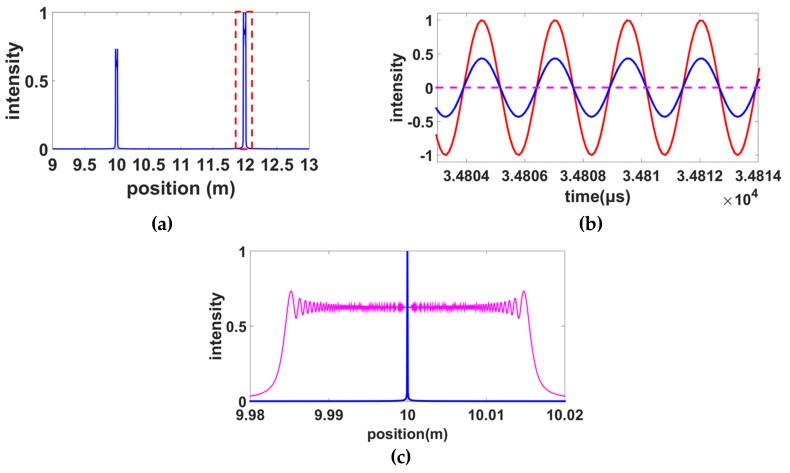
(**a**) Two reflection peaks in the spatial domain broadened by the nonlinearity tuning of the laser. (**b**) The interference patterns over a period of time. The blue curve is the ideal interference pattern in the temporal domain. The red curve is the signal recovered by the band-pass filtered signal shown in (a). (**c**) The pink and blue curves are the first reflection peak without and with the nonlinearity correction using the self-reference method. All intensities are normalized.

Two reflection points on the measured fiber were simulated and their OPDs (roundtrip) were 20 m and 24 m. The second reflection point was the reference point used to correct nonlinearity. Figure 7a shows the OFDR trace in the spatial domain. It can be seen that the peaks were broadened centered at 10 and 12 m, resulting from the tuning nonlinearity of the laser. The spatial resolution was, therefore, deteriorated. In Figure 7b, the blue curve represents the ideal interference pattern in the temporal domain, which is the interference between the ideal reflection at the position of 24 m and the local light. The red curve represents the result after the inverse fast Fourier transformation (IFFT) operation (before the phase calculation) shown in Figure 6. It can be seen that the recovered interference pattern had the same phase as the ideal interference pattern. Therefore, the self-reference method had the same effect as the conventional auxiliary interferometer shown in Figure 3. Figure 7c compares the results with and without nonlinearity correction using the self-reference method. It can be seen that, after the nonlinearity correction, the Fourier transform-limited spatial resolution can be achieved.

### 3.3. Experiment

**Figure 8:**
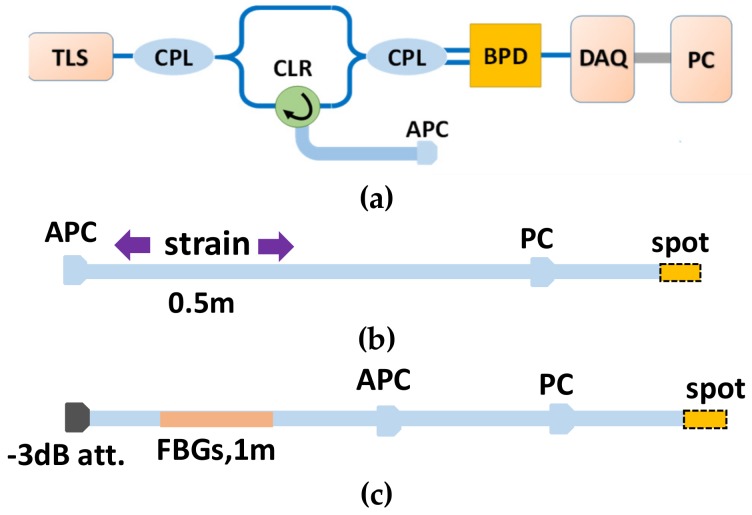
OFDR system using the self-reference method for nonlinearity correction. (**a**) Basic OFDR interrogation system. (**b**) Configuration 1, composed of a single-mode fiber. (**c**) Configuration 2, where the fiber part between the attenuator and the APC connector is a Ge-doped fiber with 1-m-long dense weak FBG arrays inscribed in the middle. The end of the fiber is knotted for reducing of the reflectivity. A PC connector is used for the self-reference reflectivity point.

The OFDR system using self-reference for the nonlinearity correction was similar to the system shown in Figure 3. However, as shown in Figure 8a, only the main interferometer was kept. The TLS swept from 1540 nm to 1560 nm, and its nominal tuning speed was 40 nm/s. The sampling rate of the data acquisition card (DAQ) was10 MS/s. Two configurations were investigated. The first is shown in Figure 8b. All segments were composed of a single-mode fiber. The second is shown in Figure 8c. The fiber part between the −3-dB attenuator and the APC connector was a Ge-doped fiber with 1-m-long dense weak FBG arrays inscribed in the middle. Each single FBG had a length of 8 mm and a gap of 2 mm. The weak FBG arrays were made using a phase mask and UV light. The nominal central wavelength was 1550 nm. A 3 dBattenuator was inserted before the sensing segment to reduce the incident light power. The PC connectors in the two configurations were used as the reference reflectivity point in the process of nonlinearity correction.

**Figure 9:**
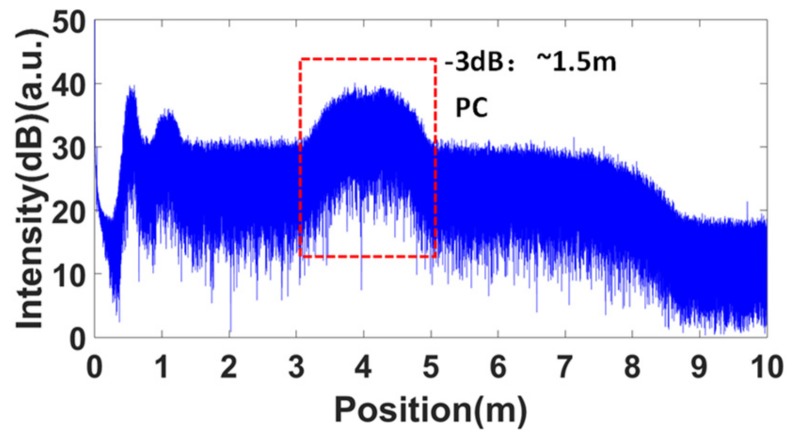
Measured OFDR trace without nonlinearity correction. The FWHMof the PC connector is about 1.5 m.

**Figure 10:**
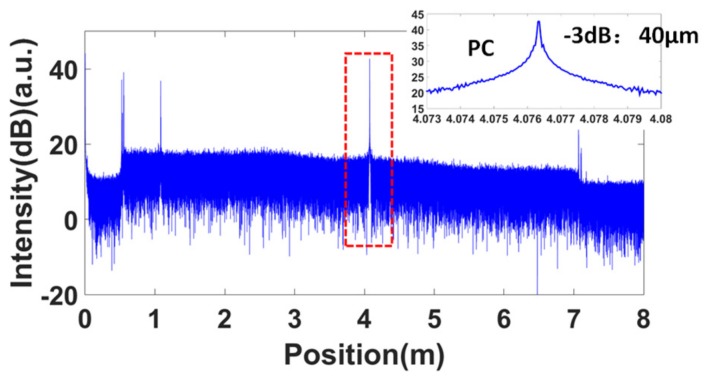
OFDR trace of configuration 1 with nonlinearity correction. The inset is the reflection of the PC connector.

**Figure 11:**
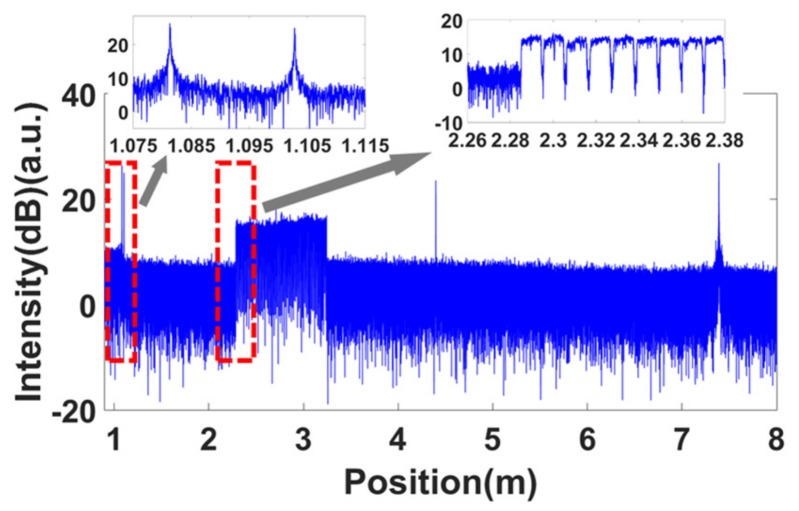
OFDR trace of configuration 2 with nonlinearity correction.

Firstly, the spatial resolution tests for these two configurations were performed. The equal phase intervals in the self-reference for the nonlinearity correction were all set to π/2. Figure 9 shows the measured OFDR trace of configuration 1 without nonlinearity correction. The FWHM of the PC connector was about 1.5 m. Then, the band-pass filter shown in the red dashed box in Figure 9 was used to extract the optical phase information of the laser. The correction process followed the process shown in Figure 6. The corrected OFDR trace is shown in Figure 10. The inset is the reflection of the PC connector. The FWHM of the PC connector was about 40 μm, which was equal to the Fourier transform-limited spatial resolution. Figure 11 shows the measured OFDR trace of configuration 2 after nonlinearity correction. The left inset in Figure 11 is the attenuator. The distance between two APC reflectivity planes was about 2 cm, which is in agreement with the practical value. The right inset in Figure 11 is the beginning of the FBG arrays. It can be seen that the FBG was about 10 dB higher than the Rayleigh scatter level. The interval and reflectivity strengths were even. From the results of the spatial resolution tests, the nonlinearity correction method using self-reference was effective. The structures on the measured fiber were all distinguished and measured with high accuracy and resolution.

**Figure 12:**
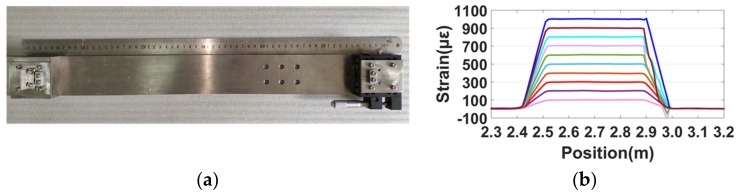
(**a**) Linear displacement stage to apply a certain strain to the fiber. (**b**) The distributed strain upon increasing strain from 100 με to 1000 με with 100-με intervals.

Then, the distributed sensing tests were implemented. The distributed strain was measured using the conventional demodulation method demonstrated in References [1,12]. The nonlinearity correction was achieved using the self-reference method. The gauge length was set to 1 cm. The stretching part was a single-mode fiber with acrylate coating as shown in Figure 8b. The measured fiber was stretched by a nanometer stage as shown in Figure 12a. The device could generate a standard strain calculated by ε=ΔL/L, where ΔL is the elongation length of the fiber and L is the original length. The distributed strains are shown in Figure 12b. Thus, it is certain that the self-reference method does not influence the classical distributed strain demodulation in OFDR.

## 4. Conclusions

In summary, we developed methods for tuning nonlinearity correction in an OFDR system from the aspect of data acquisition and post-processing. Based on their principles, these two methods both took advantage of the auxiliary interferometer information (in the self-reference method, a PC-constituted interferometer served as the auxiliary interferometer) to find the equal-spacing frequency position. The difference was that the former triggered the acquisition only at the position of zero-crossing, while the latter extracted the phase information to obtain the continuous phase changing of the laser. Therefore, in the second method, a smaller frequency interval can be set, and this makes it possible to achieve nonlinearity correction for a longer measurable range. Another difference between these two nonlinearity correction methods is that the correction method implemented by the hardware is high-speed and in real time. The correction using post-processing is not in real time, although it can approach real time with the usage of high-performance computing equipment. The advantage of the self-reference method lies in that, compared to the conventional post-processing method, the self-reference method can reduce the hardware and data burden for the system, and it is expected to have potential value in system integration and miniaturization.
